# Editorial: Mitochondrial OXPHOS System: Emerging Concepts and Technologies and Role in Disease

**DOI:** 10.3389/fcell.2022.924272

**Published:** 2022-05-19

**Authors:** Erika Fernández-Vizarra, Sylvie Callegari, Glòria Garrabou, David Pacheu-Grau

**Affiliations:** ^1^ Istituto Veneto di Medicina Molecolare (VIMM), Padova, Italy; ^2^ Dipartimento di Scienze Biomediche, Università di Padova, Padova, Italy; ^3^ Walter and Eliza Hall Institute of Medical Research, Parkville, VIC, Australia; ^4^ Department of Medical Biology, University of Melbourne, Melbourne, VIC, Australia; ^5^ Muscle Research and Mitochondrial Function Laboratory, Internal Medicine Department-Hospital Clínic of Barcelona, Cellex-IDIBAPS, Faculty of Medicine and Health Sciences, University of Barcelona, Barcelona, Spain; ^6^ Centro de Investigaciones Biomédicas en Red de Enfermedades Raras (CIBERER), Madrid, Spain; ^7^ Departamento de Bioquímica, Biología Molecular y Celular, Universidad de Zaragoza, Zaragoza, Spain; ^8^ Instituto de Investigación Sanitaria (IIS) de Aragón, Zaragoza, Spain

**Keywords:** mitochondria, OXPHOS, mtDNA, mitochondrial proteome, mitochondrial dysfunction, mitochondrial quality control

Mitochondria are eukaryotic organelles responsible for generating the main bulk of ATP, the cellular energy currency, via the process of oxidative phosphorylation (OXPHOS). The OXPHOS system is unique because it comprises subunits of dual genetic origin, encoded in the mitochondrial and the nuclear genomes. Therefore, to form the multimeric membrane-bound complexes responsible for this energy production process, proteins translated inside the organelle must be assembled in coordination with those expressed in the cytosol and imported into mitochondria by using a sophisticated import and translocation machinery. The idea that the OXPHOS system plays a role not only in ATP production but also in regulating many physiological and pathological processes has been emerging for more than 10 years. Recent evidence points to the existence of intricate quality control systems that guarantee the functionality of mitochondria, as well as the interactions of intramitochondrial and extramitochondrial factors that ultimately influence mitochondrial bioenergetics. For these reasons this Research Topic is a timely release, covering emerging concepts relating to the structure, function, and regulation of the OXPHOS system. In addition, the Research Topic also presents novel technological approaches to unravel the yet unknown intricacies involving this group of protein complexes as well as new mechanisms or pathways linking OXPHOS dysfunction and pathological states.

This Research Topic comprises thirteen contributions, including nine reviews, one perspective and three original research articles that highlight relevant new aspects influencing mitochondrial OXPHOS fitness. Guitart-Mampel et al. analyze the role of inorganic polyphosphate (polyP) in bioenergetics by using wild-type and MitoPPX SH-SY5Y (cells enzymatically deprived of mitochondrial polyP) via a thorough analysis of the changes in cell physiology, proteome and metabolome. Two key metabolic mitochondrial pathways (OXPHOS and TCA cycle) were affected by deficiency in polyP. This observation could be linked to a regulatory role of polyP in processes altered in neurodegenerative disorders, such as reactive oxygen species (ROS) production, apoptosis, inflammation, or energy metabolism. Illescas et al. explore mitochondrial function within a broader cellular context, depicting how cytoskeleton dynamics is related not only to mitochondrial trafficking, dynamics, and apoptosis but also to biogenesis and metabolism. Interestingly, they report novel findings of actin filaments located outside and inside mitochondria and the relevance of gelsolin (one of the most abundant actin-binding proteins) to mitochondrial function. Other articles in the Research Topic offer an updated view of key molecular pathways inside mitochondria. Padavannil et al. explore the intriguing question of why accessory subunits of Complex I (CI) have been added during evolution to the fourteen conserved core subunits. To explain the evolutionary and functional reasons for these additions, they used a combination of available structural information from CI assembly, subunit knockout, knockdown, and mutagenesis, and clinical studies. As a result of this analysis, they propose more sophisticated molecular functions for each of the accessory subunits, including, for example, assembly coordination, scaffold for protein localization to the cristae, response to ROS generation and sensors for energy supply/output. Eaglesfield and Tokatlidis provide an updated overview concerning the structure and mechanisms of protein insertion into mitochondria, highlighting the most exciting features of the various molecular machineries conducting protein translocation and insertion (including the TOM, MIM, SAM, TIM22 and TIM23 complexes, and OXA1). Den Brave et al. summarized the most recent and compelling insights on protein quality control mechanisms at the mitochondrial surface. They describe the current molecular pathways by which cells can survey and cleanup the mislocalization of mitochondrial proteins and protein blockages of the mitochondrial import channel in order to ensure optimal mitochondrial function.

An important focus of this Research Topic has been to highlight new experimental approaches that can be implemented to uncover different aspects of mitochondrial biology. Mitochondrial disorders affect various tissues and organs, and developing physiologically relevant cellular models is critical to understanding the basis of their pathology. Pavez-Giani and Cyganek present the latest advances in the use of human induced pluripotent stem cells to study mitochondrial cardiomyopathies. They analyzed different reported iPSC-CM models used to study a range of mitochondrial genetic mutations and their characteristics. They additionally describe the general limitations of this approach and different strategies to overcome them. Cabrera-Orefice et al. summarize the most relevant aspects of Complexome Profiling. This resolutive approach combines the separation of membrane complexes by native electrophoresis, size exclusion chromatography, or density gradient centrifugation with proteomic mass spectrometry analysis. This technique has proven its usefulness in evaluating the inventory, abundance, and arrangement of OXPHOS Complexes and related factors in health and disease. The authors give examples of different uses for this technical approach and discuss the limitations, improvements, and the use of Complexome Profiling to complement, for example, structural methods. Finally, Robinson et al. present an elegant original research article illustrating the application of carbonate extraction of mitochondrial membrane proteins, coupled to mass spectrometry, to investigate OXPHOS structural defects. In this way, they generated membrane association profiles for >800 mitochondrial proteins and clustered them into 6 different groups based on resistance to carbonate extraction at different pH Using Complex III (CIII) knockout cell lines as an example, they used this technique to assess the destabilization of protein associations within the mitochondrial membranes induced by CIII assembly defects.

The last group of articles uncovers new concepts related to mitochondrial dysfunction and disease. Zhang et al. link mitochondrial dysfunction and severe liver disease. Their review discusses how liver dysfunction impacts mitochondrial fitness and consequently which mitochondrial parameters could be used as a hallmark of liver disease. They further define novel methodologies for assessing mitochondrial function in this context and propose strategies to reverse the metabolic reprogramming observed in advanced liver disease. In their perspective article, Flønes and Tzoulis evaluate the role and influence of OXPHOS in idiopathic Parkinson’s disease (PD) neurodegeneration. They consider different aspects of OXPHOS dysfunction in PD patients to enlighten the nature of this defect in the brain. More specifically, they explore the discrepancies found in the OXPHOS phenotypes of PD patients, possible molecular causes for the defects, links to other known hallmarks of PD, such as alpha-synuclein, and the potential downstream impact of OXPHOS dysfunction in PD pathology. Chowdhury et al. summarize the most recent insights into the role of released mitochondrial nucleic acids and immune system activation. They provide a comprehensive review of the main pathways involved in nucleic acid release from mitochondria, sensing mechanisms, and the consequences of these molecular processes on human health. They point out how mitochondrial RNA and DNA release may play an essential role in critical diseases such as autoimmune disorders and neuroinflammatory diseases. Interestingly, mitochondrial gene expression requires approximately 25% of the mitochondrial proteome, highlighting the complexity and relevance of this process for mitochondrial physiology. Wang et al. present a thorough review analyzing the connection between mitochondrial translation and disease. They review the different molecular steps in the process and the known mutations that affect these steps. Furthermore, they discuss the possibility of different regulatory mechanisms mediated by microRNAs, as well as the coordination between cytosolic and mitochondrial translation. Finally, Jennings et al. contributed an original research article describing the molecular consequences of DNAJC3 mutations associated with a multisystemic neurological disorder and diabetes. Disruption of this Endoplasmic Reticulum (ER) BiP co-chaperone leads to alteration of lipid and cholesterol metabolism, resulting in ER stress, activation of the unfolded protein response (UPR), *β*-amyloid accumulation, and ultimately an alteration of the OXPHOS system. Interestingly, the abundance of OXPHOS subunits and, concomitantly, mitochondrial respiration rates were increased in these mutants, indicating a possible pro-survival mechanism triggered by ER-stress.

In conclusion, this Research Topic of articles summarizes and describes novel molecular aspects of mitochondrial fitness, state-of-the-art technologies to study mitochondrial organization, and novel associations of mitochondrial dysfunction to diseases. It provides an update on the most relevant insights into mitochondrial pathophysiology, setting the path to bringing discoveries into current experimental and clinical practices.

